# Exploring the Relationship Between Brain-Derived Neurotrophic Factor Haplotype Variants, Personality, and Nicotine Usage in Women

**DOI:** 10.3390/ijms26157109

**Published:** 2025-07-23

**Authors:** Dominika Borowy, Agnieszka Boroń, Jolanta Chmielowiec, Krzysztof Chmielowiec, Milena Lachowicz, Jolanta Masiak, Anna Grzywacz, Aleksandra Suchanecka

**Affiliations:** 1 Independent Laboratory of Behavioral Genetics and Epigenetics, Pomeranian Medical University in Szczecin, Powstańców Wielkopolskich 72 St., 70-111 Szczecin, Poland; 68103@student.pum.edu.pl (D.B.); anna.grzywacz@pum.edu.pl (A.G.); 2 Department of Clinical and Molecular Biochemistry, Pomeranian Medical University in Szczecin, Powstańców Wielkopolskich 72 St., 70-111 Szczecin, Poland; agnieszka.boron@pum.edu.pl; 3 Department of Hygiene and Epidemiology, Collegium Medicum, University of Zielona Góra, 28 Zyty St., 65-046 Zielona Góra, Poland; j.chmielowiec@inz.uz.zgora.pl (J.C.); k.chmielowiec@inz.uz.zgora.pl (K.C.); 4 Department of Psychology, Gdansk University of Physical Education and Sport, 80-336 Gdansk, Poland; milena.lachowicz@awf.gda.pl; 5 Second Department of Psychiatry and Psychiatric Rehabilitation, Medical University of Lublin, 1 Głuska St., 20-059 Lublin, Poland; jolanta.masiak@umlub.pl

**Keywords:** *BDNF*, nicotine, NEO Five-Factor Inventory, personality, anxiety

## Abstract

Brain-derived neurotrophic factor (BDNF) is associated with nicotine use behaviours, the intensity of nicotine cravings, and the experience of withdrawal symptoms. Given the established influence of sex, brain-derived neurotrophic factor variants, personality traits and anxiety levels on nicotine use, this study aimed to conduct a comprehensive association analysis of these factors within a cohort of women who use nicotine. The study included 239 female participants: 112 cigarette users (mean age = 29.19, SD = 13.18) and 127 never-smokers (mean age = 28.1, SD =10.65). Study participants were examined using the NEO Five-Factor Inventory and the State–Trait Anxiety Inventory. Genotyping of rs6265, rs10767664, and rs2030323 was performed by real-time PCR using an oligonucleotide assay. We did not observe significant differences in the distribution of either genotype or allele of rs6265, rs10767664 and rs2030323 between groups. However, compared to the never-smokers, cigarette users scored significantly lower on the Agreeableness (5.446 vs. 6.315; *p* = 0.005767; d_Cohen’s_ = 0.363; η^2^ = 0.032) and the Conscientiousness (5.571 vs. 6.882; *p* = 0.000012; d_Cohen’s_ = 0.591; η^2^= 0.08) scales. There was significant linkage disequilibrium between all three analysed polymorphic variants—between rs6265 and rs10767664 (D′ = 0.9994962; *p* < 2.2204 × 10^−16^), between rs6265 and rs2030323 (D′ = 0.9994935; *p* < 2.2204 × 10^−16^) and between rs10767664 and rs20330323 (D′ = 0.9838157; *p* < 2.2204 × 10^−16^), but the haplotype association analysis revealed no significant differences. While our study did not reveal an association between the investigated brain-derived neurotrophic factor polymorphisms (rs6265, rs10767664 and rs2030323) and nicotine use, it is essential to acknowledge that nicotine dependence is a complex, multifactorial phenotype. Our study expands the current knowledge of *BDNF* ’s potential role in addictive behaviours by exploring the understudied variants (rs10767664 and rs2030323), offering a novel contribution to the field and paving the way for future research into their functional relevance in addiction-related phenotypes. The lower Agreeableness and Conscientiousness scores observed in women who use nicotine compared to never-smokers suggest that personality traits play a significant role in nicotine use in women. The observed relationship between personality traits and nicotine use lends support to the self-medication hypothesis, suggesting that some women may initiate or maintain nicotine use as a coping mechanism for stress and negative affect. Public health initiatives targeting women should consider personality and psychological risk factors in addition to biological risks.

## 1. Introduction

According to the World Health Organisation (WHO), in 2022, the prevalence of nicotine use among the global population was 17%. Twenty-nine per cent of males were smokers, compared to five per cent of females. The observed decline is evidenced by the 2007 statistics, which showed that 22.8% of the global population were smokers, including 38% of men and 8% of all women [1]. Currently, the new methods of nicotine use (vaping, e-cigarettes and nicotine pouches) are on the rise. Following the increase in popularity of disposables, vaping has become increasingly prevalent among young adults, including those who have never smoked regularly. Consequently, the downward trends in inhaled nicotine use have been reversed. Recent data indicate a marginal increase in cigarette smoking among older age groups; conversely, the prevalence of smoking has continued to decline among 18- to 24-year-olds, the age group that has exhibited the most significant increase in disposable vaping [2]. Nicotine use is the cause of many avoidable mortalities, attributable to its toxicity. The most common one is lung cancer, a major health issue associated with nicotine use. Nevertheless, other diseases must be considered, including various forms of cancer, cardiovascular disease, chronic obstructive pulmonary disease, myocardial infarction and coronary artery disease [3,4].

Substance dependence (SD) involves a pattern of compulsive substance-seeking and use that persists despite the awareness of negative consequences [5]. A key aspect of SD is the capacity of psychoactive substances to induce neurobiological changes, ultimately shaping behaviour. The specific neuroadaptations and behavioural manifestations depend on a complex interplay of factors, including the pharmacological properties of the substance, the patterns of exposure (frequency and duration) and individual characteristics such as metabolic capacity, sensitivity to the substance’s effects and pre-existing psychiatric conditions [6]. Nicotine, the primary active substance in tobacco products, stimulates dopamine release in the midbrain, contributing to its addictive potential [7].

Brain-derived neurotrophic factor (BDNF), the most abundant neurotrophic factor in the brain, plays a crucial role in various aspects of neuronal function, including neuronal development and maturation, neuroregeneration, neuroprotection, synapse formation and plasticity, as well as in cognitive processes such as learning and memory [8,9,10,11]. BDNF binds selectively to the tyrosine kinase receptor B (TrkB) [12]. However, it can also interact with the p75 neurotrophin receptor (p75NT), which has been implicated in promoting apoptosis within the nervous system [8,13]. The *BDNF* gene is primarily expressed in dopaminergic neurons within the ventral tegmental area and nucleus accumbens [8,14]. As a member of the neurotrophin family [12,13], BDNF is initially synthesised in the central nervous system (CNS) as a precursor molecule, pre-pro-BDNF [10]. Calcium-permeable glutamate receptors, like N-Methyl-D-aspartate (NMDA) receptors, and voltage-gated Ca^2+^ channels regulate *BDNF* gene transcription by binding transcription factors to promoter regions [10]. The human *BDNF* gene comprises 11 exons, with alternative splicing of transcripts contributing to tissue-specific variations [15].

Nicotine dependence (ND) is associated with compulsive smoking [8,16,17] due to neuroadaptive and psychological changes caused by repeated nicotine use, which are linked to learning and memory processes, mainly through the activation of the brain’s reward circuitry [18,19]. Chronic nicotine administration leads to an upregulation of *BDNF*, accompanied by increased dopamine (DA) release and heightened dopamine receptor 3 (*D3R*) expression [20,21]. In the mesolimbic dopaminergic system (MDS), nicotine’s effect starts with binding to the nicotinic acetylcholine receptors (nAChRs) in the ventral tegmental area (VTA), prompting DA release into the nucleus accumbens (NAc) [22,23,24]. Nicotine increases DA release through α4β2 nAChRs and α7 nAChRs located on VTA dopaminergic neurons, activating dopamine D1 and D2 receptors in the NAc [25]. The activation of these receptors initiates a signalling cascade involving MEK phosphorylation and increased ERK1/2 activity, leading to CREB phosphorylation [26,27], a step that is critical for nicotine’s reinforcing properties [28]. The current understanding suggests that nicotine modulates *BDNF* expression in the NAc by activating nAChRs and boosting DA release [8]. Subsequently, released DA binds to DRs, triggering downstream signalling pathways that enhance the transcription of the *BDNF* gene [8]. Additionally, dopamine release within the NAc, triggered by nicotine, results in positive reinforcement, a mechanism particularly prominent in the early stages of ND [8,29].

The *BDNF* gene is studied in the context of nicotine use behaviours, the intensity of nicotine cravings and the experience of withdrawal symptoms [30,31,32,33]. The most widely studied *BDNF* SNP rs6265, located within an exon of the gene, results in a valine-to-methionine substitution at the 66th codon (Val66Met) of the BDNF protein [8,34]. This change arises from a cytosine-to-tyrosine substitution on the forward strand of the DNA [35]. Rs 6225 has been linked to an elevated risk of suicidal behaviour and heroin dependence [36,37]. It influences susceptibility to stress and a range of stress-related and stress-inducible neuropsychiatric endophenotypes in both humans [38] and rodent models [39]. This has led to the development of a BDNF stress-sensitivity hypothesis [40], which suggests that the disruption of normal BDNF function by factors such as the Val66Met variant increases sensitivity to stress and, consequently, vulnerability to stress-related disorders. BDNF’s role in plasticity may contribute to both the maladaptive encoding of fear and the adaptive plasticity observed during extinction learning, which can suppress fear responses [41,42,43]. Therefore, factors that regulate BDNF availability, such as the Val66Met polymorphism, may orchestrate sensitivity to stress, trauma and the risk of developing stress-induced disorders [40]. Evidence suggests that variations in brain-derived neurotrophic factor activity are gender-dependent and modulated by sex hormones [44]. The rs10767664 and rs2030323 are located within the introns of the *BDNF* gene [45,46]. Rs10767664 involves a substitution of tyrosine by adenine [46], while rs2030323 involves a substitution of adenine by thymine [45]. Rs10767664 has been associated with coronary artery disease-related depression and antidepressant response [25,47], suicidal behaviour [48] and heroin addiction [49]. Rs2030323 has been associated with the Harm Avoidance personality trait [50].

The Big Five [51,52] is a model of personality that includes five traits: Extroversion, Neuroticism, Agreeableness, Openness to Experience and Conscientiousness. Extraversion is connected to assertiveness and social skills; Neuroticism is the tendency to lack emotional stability; Agreeableness is associated with politeness; Openness is sensitivity to art, broad interests and seeking novelty; and Conscientiousness is being task-focused, neat and orderly [53]. The model is widely used in substance dependence and addiction studies. Generally, the traits associated with SD are high Neuroticism and low Conscientiousness and Agreeableness [54,55]. The traits that are approach-related, such as Extraversion, Novelty seeking and Impulsivity, and avoidance-related, such as Neuroticism and Harm avoidance, are most associated with smoking behaviour [56]. Additionally, low Conscientiousness or Agreeableness or high levels of Extraversion during childhood were reported as a predicting factor of smoking in adulthood [57,58].

Anxiety, as a state and as a trait, is frequently investigated in the context of substance dependence. A commonly used tool for measuring both aspects of anxiety is the State–Trait Anxiety Inventory (STAI) [59,60]. Trait anxiety is characterised by a heightened perception of threat and an enhanced capacity to recall threatening situations, often resulting in difficulties with adapting to new information and avoiding potentially threatening circumstances [61]. State anxiety, on the other hand, reflects the subjective experience at a specific point in time [61]. Importantly, high levels of anxiety are reported as related to substance dependence [62].

Women are relatively more likely to use tobacco, struggle more with smoking cessation and experience less success with cessation therapies compared to men [63,64,65,66]. This leads to a higher incidence of tobacco-related health problems in women [67,68,69,70]. Clinical findings indicate that women are more susceptible to anxiety disorders [71,72,73,74] and are more likely to use smoking as a coping mechanism for stress [66,75,76,77,78]. During attempts to quit smoking, women report higher levels of anxiety than men [79,80,81], and frequently attribute their continued tobacco use and relapse to the perceived stress-reducing effects of nicotine [65,66,75,76,77]. These observations are supported by preclinical studies in rodents, which demonstrate that females exhibit more pronounced stress responses during nicotine withdrawal compared to males [82]. Stress may be a key factor influencing both the initiation of tobacco use and relapse patterns in women. Additionally, recent data from 2024 [83] suggest a shift in smoking patterns among women of reproductive age. Over the past decade, there has been an increase in smoking prevalence among women in higher socioeconomic groups. Furthermore, across all social groups, there has been a significant rise in the popularity of vaping, along with a transition from manufactured cigarettes to hand-rolled cigarettes among women who smoke. These changes are more pronounced in this demographic than in the general adult population.

The central hypothesis of this study was that genetic variation in the *BDNF* gene, specifically three polymorphisms (rs6265, rs10767664 and rs2030323), may be associated with nicotine use in women. Additionally, it was hypothesised that personality traits and anxiety levels might also be related to nicotine use. Hence, given the underrepresentation of women in nicotine dependence research and the growing recognition of sex-specific influences on addiction, this study focuses on investigating the interplay between *BDNF* polymorphisms, personality traits and nicotine use in a female-only sample.

## 2. Results

### 2.1. The Hardy–Weinberg Equilibrium

The Hardy–Weinberg equilibrium is based on the principle that genotype frequencies are stable for autosomal variants in the absence of altering or disturbing factors [84]. In both groups, the frequency distribution of rs6265, rs10767664 and rs2030323 alleles was under the Hardy–Weinberg equilibrium. The results are presented in Table 1, Table 2 and Table 3, respectively. The number of samples differs in Table 1, Table 2, Table 3, Table 4, Table 5 and Table 6 due to missing genotypes.

### 2.2. Association of BDNF rs6265, rs10767664 and rs2030323 Genotypes and Alleles with Cigarette Smoking

There was no significant difference in the distribution of *BDNF* rs6265 genotypes. The distribution of rs6265 genotypes in the case group compared to the control group was A/A 0.0089 vs. A/A 0.0079; A/G 0.2232 vs. A/G 0.2441; G/G 0.7679 vs. G/G 0.748, χ^2^ = 0.1495, *p* = 0.92796. There was also no significant difference between groups in the frequency of rs6265 alleles. The allele distribution in the case group compared to the control group was A 0.1205 vs. A 0.1299; G 0.8795 vs. G 0.8701, χ^2^ = 0.0955, *p* = 0.7573 (Table 4).

There was no significant difference in the distribution of *BDNF* rs10767664 genotypes. The distribution of rs10767664 in the case group compared to the control group was A/A 0.009 vs. A/A 0.0165; A/T 0.2703 vs. A/T 0.314; T/T 0.7207 vs. T/T 0.6694, χ^2^ =0.8513, *p* = 0.65336. There was also no significant difference between groups in the frequency of rs10767664 alleles. The allele distribution in the case group compared to the control group was A 0.1441 vs. A 0.1736; T 0.8559 vs. T 0.8264, χ2 = 0.7471, *p* = 0.3874 (Table 5).

There was no significant difference in the distribution of *BDNF* rs2030323 genotypes. The rs2030323 genotype distribution in the case group compared to the control group was G/G 0.7182 vs. G/G 0.6529; G/T 0.2727 vs. G/T 0.3058; T/T 0.0091 vs. T/T 0.0413, χ^2^ = 2.8807, *p* = 0.23684. There was also no statistically significant difference between groups in the frequency of rs2030323 alleles. The allele distribution in the case group compared to the control group was G 0.8545 vs. G 0.8058; T 0.1455 vs. T 0.1942, χ^2^ = 1.9328, *p* = 0.1644. The results are presented in Table 6.

### 2.3. Personality Trait Assessment by the NEO Five-Factor Inventory and State–Trait Anxiety Inventory

Cigarette users, when compared to the never-smokers, scored significantly lower on the Agreeability (5.446 vs. 6.315; *p* = 0.005767) and the Conscientiousness (5.571 vs. 6.882; *p* = 0.000012) scales. There were no significant differences in the Neuroticism (5.893 vs. 5.622; *p* = 0.255490), Extraversion (5.616 vs. 5.299; *p* = 0.239026) and Openness (5.411 vs. 5.457; *p* = 0.798012) scores. Eta-squared and Cohen’s d indicated a small effect size for Agreeableness (0.363; ~3% of the variance explained) and a moderate effect size for Conscientiousness (0.591; ~8% of the variance explained). The results are presented in Table 7.

There was no significant statistical difference in trait (5.902 vs. 5.606; *p* = 0.174650) and state (5.598 vs. 5.512; *p* = 0.653407) anxiety means obtained by cigarette users and never-smokers. The results are presented in Table 8.

### 2.4. Linkage Disequilibrium and Haplotype Association Analysis

There was significant linkage disequilibrium (LD) between all three analysed polymorphic variants—between rs6265 and rs10767664 (D′ = 0.9994962; *p* < 2.2204 × 10^−16^), between rs6265 and rs2030323 (D′ = 0.9994935; *p* < 2.2204 × 10^−16^) and between rs10767664 and rs20330323 (D′ = 0.9838157; *p* < 2.2204 × 10^−16^). The results are presented in Table 9.

The haplotype association analysis revealed no significant differences. The frequencies of the GAT, AAT and GTG haplotypes in the control group were 0.03356, 0.13866 and 0.81087, while in the case group, they were 0.02273 (*p* = 0.47669), 0.12273 (*p* = 0.59990) and 0.85455 (*p* = 0.20167), respectively. The results are presented in Table 10.

## 3. Discussion

In the present study, we aimed to perform a comprehensive association analysis of brain-derived neurotrophic factor variants, personality traits, anxiety levels and nicotine use in a group of women and never-smoking controls. We performed an exploratory study rather than a hypothesis-confirmatory one. We did not pre-specify directional hypotheses for each individual SNP or personality trait. Instead, we took a multidisciplinary approach, integrating genetic, psychological and behavioural data.

The brain-derived neurotrophic factor is associated with numerous mental disorders. The rs6265 variant modifies the distribution of BDNF to neuronal dendrites and its activity-dependent secretion [85,86], despite not affecting the mature protein sequence. Met allele carriers have smaller hippocampal volumes [87,88] and poorer performance on hippocampus-dependent memory tasks [89]. The Met allele, associated with reduced BDNF availability, is therefore considered a risk factor for psychopathology, including higher neuroticism and anxiety, depression and suicide risk [90,91,92,93]. Furthermore, the peripheral and brain BDNF levels differ between smokers and non-smokers [94]. While some studies indicate lower peripheral BDNF levels in smokers [30,32], others suggest higher levels [31,94]. BDNF levels have also been shown to change during smoking cessation [32,94]. Interestingly, Jamal et al. [31] found that smokers (with and without ND) had significantly higher levels of serum BDNF compared to never-smokers and former smokers. This effect was independent of the number of cigarettes smoked daily, but the number of smoking years was associated with the serum level. Additionally, no association was found between the Val66Met polymorphism and *BDNF* serum levels.

Our results did not reveal any significant associations between the investigated *BDNF* polymorphisms (rs6265, rs10767664 and rs2030323) and nicotine usage in the analysed sample of women. However, we found significant linkage disequilibrium between the analysed variants, which allowed us to perform haplotype association analysis of rs6265-rs10767664–rs2030323, which rendered insignificant results. Research on the relationship between the functional *BDNF* Val66Met polymorphism and nicotine dependence has yielded conflicting results. While some studies suggest a link between rs6265 and nicotine dependence [95,96,97], many others have failed to confirm this association [30,98,99,100]. These inconsistencies may be due to a variety of factors, including population differences. Some studies have linked this SNP to earlier smoking initiation [101,102] and aspects of smoking behaviour [31,33,98]; these associations have not been consistently replicated across different ethnic groups [103,104]. Korhonen et al. [101] reported an association between rs6265 and smoking, with the GA and GG genotypes being more prevalent in smokers than in controls. However, Nedic et al. [105] found no significant association between the Val66Met genotypes or alleles and alcohol dependence in either male or female subjects. Similarly, Novak et al. [106] found no direct association between rs6265 and smoking in schizophrenia patients. However, haplotype analysis revealed that the ACCG haplotype of the rs6265, rs11030104, rs2049045 and rs7103411 block was associated with an increased risk of nicotine dependency. In a study on heroin dependence, Meng et al. [107] found no overall association with rs6265 allele frequencies. Still, they reported that the AA genotype was linked to an earlier onset of heroin dependence compared to the GG genotype. A significant association of the rs6265 was found by Strońska-Puta et al. [108] when comparing both the individuals undergoing addiction treatment for the first time and individuals after a relapse to a control group. Similarly, Boroń et al. [109] reported a significant association between rs6265 and alcohol use disorder in females. Rs10767664 has been linked to the modulation of both mood and anxiety in human and mouse models [110]. In contrast, rs2030323 has been associated with the Conscientiousness trait [111]. Interestingly, rs10767664 has also been implicated in metabolic processes, with reports linking it to diabetes mellitus, insulin resistance and insulin levels [112,113]. Similarly, rs2030323 has also been associated with caloric intake [114].

The present study investigated differences in personality traits, assessed using the NEO-FFI, between smokers and a control group. The results indicated statistically significant differences in Agreeableness and Conscientiousness, with cigarette users scoring lower on both dimensions than the control group. The observed differences fell within the small to moderate range of effect. Prior research suggests that even minor variations in these traits can influence behavioural tendencies over time, particularly in relation to self-regulation and decision-making under stress [115]. Therefore, the observed personality differences may reflect subtle but functionally relevant predispositions that contribute to nicotine use behaviour in women. The current results partially align with our previous study regarding personality dimensions in nicotine users, which revealed that the cigarette users had significantly higher Extraversion scores and lower Openness, Agreeableness and Conscientiousness scores [116]. Our team’s study regarding e-cigarette users [117] also yielded similar results regarding Conscientiousness, with e-cigarette users scoring higher on the Conscientiousness scale and lower on the Neuroticism scale (not significant after Bonferroni correction), as well as lower on the Extraversion scale. Rass et al. [118] found that both daily and non-daily smokers had lower scores of Conscientiousness than non-smokers. Although Waga et al. [119] found no significant personality differences between smokers and non-smokers, Kulkarni et al. [120] identified associations between personality and smoking behaviours within a corporate setting. Their study revealed a link between Neuroticism and nicotine dependence and further associated Extraversion and Openness with health concerns. Agreeableness and Conscientiousness were linked to social factors influencing quitting, while Extraversion and Agreeableness were related to occupational/social factors for relapse [116]. Addiction research employing the Big Five model frequently identifies higher Neuroticism in substance-dependent individuals [115,121] and those with behavioural addictions [122,123], possibly reflecting self-medication tendencies [124]. The self-medication hypothesis is based on observation of substance-dependent or behaviourally addicted individuals, who use a specific substance or action to relieve and handle unpleasant affect states and extreme emotions. Additionally, it may serve as a means of self-esteem regulation [124,125]. Conscientiousness, a trait linked to self-discipline, is generally lower in individuals with substance use disorders [115,126], particularly in more drug-dependent compared to alcohol-dependent individuals [127]. Reduced Conscientiousness has also been observed in behavioural addictions [122,123,126,128]. Findings for Agreeableness, Extraversion and Openness to experience are less consistent, varying across substance types. While some studies link low Agreeableness to substance use [55] or low Extraversion to drug use disorders [129], others report higher Openness in drug use disorders, though primarily in non-clinical samples [115,121]. Consistent with the findings presented here, our prior studies on e-cigarette users [62], polysubstance users [130,131] and individuals with cannabis use disorders [132] have revealed similar associations between personality and substance use.

Using the State–Trait Anxiety Inventory, we examined anxiety state and trait levels in nicotine users and never-smokers. The analysis yielded no significant differences between the two groups on either scale. In contrast to our results, a study by Pietras et al. [133] showed that smokers had higher scores on both the anxiety as a trait scale and anxiety as a state scale than non-smokers. Moreover, the intensity of anxiety as a trait and anxiety as a state were not correlated with the number of pack-years smoked. Additionally, smokers more often suffer from disorders like depression, schizophrenia, anxiety and personality disorders than non-smokers [18,134,135]. Moreover, anxiety disorders frequently co-occur with substance dependence and are more prevalent in families with a history of psychoactive substance use [136]. The *BDNF* gene has been associated with the underlying mechanisms of both depression and anxiety. Research indicates that the rs6265 Met allele is associated with decreased hippocampal volume and altered human hippocampal activity [89,137,138]. Given the hippocampus’s crucial role in mood regulation, it is plausible that the *BDNF* Val66Met polymorphism may influence behaviour and anxiety. However, this effect was not visible in our sample, which consisted primarily of young, healthy women, many of whom may not yet exhibit clinically significant anxiety symptoms. In contrast, studies reporting elevated anxiety [109,135] often involve older populations or individuals with comorbid psychiatric conditions. Additionally, personality traits such as Extraversion or Neuroticism in some participants may buffer against the development of heightened anxiety despite nicotine use, acting as resilience factors, as in our sample, both traits were on moderate levels.

Our study focused on women using nicotine, a deliberate choice driven by the underrepresentation of women in substance dependence research, where studies frequently prioritise male or mixed-sex populations. Recognising the established biological, environmental and societal differences between women and men who use nicotine products [139], we aimed to contribute to a more nuanced understanding of this population. The literature suggests that women often initiate smoking as a means of coping with stress and negative affect [140] and subsequently encounter greater obstacles in achieving and maintaining abstinence [141]. However, this association was not visible in our sample. The multifaceted challenges women face during cessation encompass stress, diminished motivation, weight gain anxieties, a lack of confidence in their ability to quit and a perceived absence of adequate support [142,143]. Women also report experiencing more intense withdrawal symptoms, such as anxiety and irritability, compared to their male counterparts [143]. These factors contribute to the low cessation success rates observed among pregnant women, who frequently relapse after childbirth [12]. In addition to these cessation-specific challenges, substance-dependent women often contend with a constellation of social and personal difficulties, including a lack of familial support, diminished self-worth, societal stigmatisation and therapeutic approaches that are not adequately tailored to address their distinct needs [144]. Moreover, women are disproportionately susceptible to developing various nicotine-related comorbidities, spanning cardiovascular disease to gender-specific conditions such as menstrual irregularities, cervical malignancies and reproductive impairments [141]. Additionally, the influence of brain-derived neurotrophic factor analysed in this study is known to be sex-specific, with sex hormones playing a key regulatory role [44]. A study by Solum and Handa [145] indicated that oestrogen modulates *BDNF* expression in rats. Further supporting this, Su et al. [146] demonstrated that testosterone acts as a positive regulator of *BDNF* expression, while progesterone increases BDNF production in cerebral cortex explants and glial cells.

This study has a few limitations that should be considered when interpreting its results. The analysed sample included predominantly young Caucasian women, restricting how our findings can be generalised to more diverse populations. Furthermore, we investigated only three *BDNF* polymorphisms. A more detailed approach, incorporating epigenetic analyses and gene expression, could provide a more comprehensive understanding of the *BDNF* gene’s association with nicotine use in women. While the focus on women allowed for a targeted investigation of sex-specific factors, it also limits the direct applicability of our conclusions to men. A further limitation of this study is that we did not collect information on menstrual cycle phase or hormonal contraceptive use, both of which are known to influence sex hormone levels and may modulate *BDNF* expression. We acknowledge that we did not control for several potentially confounding environmental variables, including socioeconomic status and family history of nicotine and other substance use or the smoking pattern. Finally, we acknowledge that our sample size (*n* = 239; 112 nicotine users and 127 never-smokers) has limited power to detect small to moderate genetic effects using single-SNP analyses (64–69% power for detecting an odds ratio of 1.5 for all three SNPs). Future research should address these limitations to improve our understanding of the brain-derived neurotrophic factor, personality and anxiety association with nicotine use in women. However, we would like to underline a few strengths and broader contributions of our study. The present study was designed not only to explore genetic associations but also to investigate the multifaceted interplay between genetic variation, personality traits, anxiety levels and nicotine use in women, a population often underrepresented in addiction research. We identified significant differences in personality traits (lower Agreeableness and Conscientiousness) in nicotine-using women compared to never-smokers, aligning with existing literature linking these traits to substance use. Our study contributes to the growing body of literature examining sex-specific influences. It provides a foundation for future investigations into how genetic and personality-related risk factors converge in shaping nicotine use behaviours in women.

## 4. Materials and Methods

### 4.1. Participants and Psychometric Tools

The cross-sectional case–control study included 239 female participants: 112 cigarette users (mean age = 29.19, SD = 13.18) and 127 never-smokers (mean age = 28.1, SD =10.65). A convenience sampling method was used, and participants were recruited based on availability and willingness to participate. The group included both the university students and community volunteers. Study participants were examined using the Mini International Neuropsychiatric Interview (MINI) to exclude volunteers with neuropsychiatric disorders and substance dependencies other than nicotine. The control group, i.e., the never-smokers, were volunteers who did not smoke more than one pack of cigarettes in their lives or did not use other nicotine products. Criteria for inclusion in the study: age above 18 years, using nicotine (smoking cigarettes for the case group), or not smoking more than one pack of cigarettes during lifetime (for the control group). Study exclusion criteria: diagnosis of intellectual disability, dementia, other than nicotine substance dependence or behavioural addiction; clinically significant general health condition (cardiovascular, hepatic, renal, respiratory, haematological, endocrine or neurological disease) or traumatic brain injury in the past; the presence of psychiatric disorders other than nicotine dependence.

The NEO Five-Factor Inventory (NEO-FFI) was used to assess five personality traits: Neuroticism, Extroversion, Openness to experience, Agreeableness and Conscientiousness [48]. The State–Trait Anxiety Inventory (STAI) was used to assess state anxiety, measuring feelings of anxiety and activation of the autonomic nervous system, and trait anxiety, i.e., individuals’ propensity to feel anxious [60].

The results of both inventories were presented as sten scores and converted from the raw scores to the sten scale under the Polish standards for adults according to their age and sex. A sten value of 1–2 corresponds to very low results, 3–4 to low results, 5–6 to average results, 7–8 to high results and 9–10 to very high results [147].

The study was conducted following the Declaration of Helsinki and approved by the Bioethics Committee of the Pomeranian Medical University in Szczecin (KB-0012/164/17-A, KB-006/29/2025) in the Independent Laboratory of Behavioural Genetics and Epigenetics, Pomeranian Medical University in Szczecin.

### 4.2. Laboratory Analysis

The genomic DNA was purified from 200 µL of venous blood using a commercially available protocol (QIAamp Blood DNA Mini Kit, QIAGEN, Hilden, Germany). DNA yields were determined by measuring the DNA concentration in the eluate using absorbance at 260 nm (Picodrop, Cambridge, UK). The concentration of all samples was 20–60 ng/µL and had an A260/A280 ratio of 1.7–1.9. The genotyping process was performed using real-time PCR on the LightCycler 480II instrument (Roche Diagnostics, Basel, Switzerland) with the LightSNiP (TiBMolBiol, Berlin, Germany) oligonucleotide assay under the following conditions: initial denaturation (95 °C/10 min), followed by 45 cycles: denaturation (95 °C/10 s), annealing (60 °C/10 s) and elongation (72 °C/15 s). The melting curve of the PCR products was generated with denaturation (95 °C/30 s) and cooling (40 °C/2 min), followed by heating to 75 °C at a 1.5 °C/s rate. The reaction was terminated by cooling for 30 s at 40 °C. The melting curves were obtained by plotting the fluorescence signal and temperature. The *BDNF* gene rs6265 peaks were read at 56.94 °C for the G allele and 62.83 °C for the A allele; rs2030323 peaks were read at 55.16 °C for the T allele and 59.41 °C for the G allele; rs10767664 peaks were read at 49.4 °C for the A allele and 54.96 °C for the T allele, according to the manufacturer’s protocol. According to GRCh38.p14, the location of the *BDNF* gene is NC_000011.10 (27654893..27722030) on a complementary strand [148].

### 4.3. Statistical Analysis

The concordance between the genotype and allele distribution and Hardy–Weinberg’s equilibrium (HWE) was tested using the HWE online software (https://wpcalc.com/en/equilibrium-hardy-weinberg, accessed 10 October 2024).

The NEO-FFI and STAI scores were compared between groups using the Mann–Whitney U-Test. The differences between the control and case groups in allele and genotype frequencies for rs6265, rs10767664 and rs2030323 were analysed using the chi-square test (χ^2^ test). The Mann–Whitney U-Test and the chi-square test were performed using STATISTICA 13 software (Tibco Software Inc., Palo Alto, CA, USA) for Windows 11 (Microsoft Corporation, Redmond, WA, USA).

Effect size was calculated with online software (https://www.psychometrica.de/effect_size.html, accessed 10 July 2025).

Linkage disequilibrium (LD) and the frequency of haplotypes were analysed in the R environment, version 4.3.0, using the packages haplo.stats and genetics, and further compared between groups with the χ^2^ test. In all analyses, *p* < 0.05 was considered statistically significant.

For genetic analyses, individuals with missing genotype calls for a specific SNP were excluded from that particular analysis. No imputation was performed, and all statistical tests were based on available genotype data. Variations in sample size across SNPs reflect differences in missingness between genetic variants.

## 5. Conclusions

This study aimed to investigate the relationship between *BDNF* variants, personality and nicotine use in women, a population often underrepresented in addiction research. While our study did not reveal an association between the investigated *BDNF* polymorphisms (rs6265, rs10767664 and rs2030323) and nicotine use, it is essential to acknowledge that ND is a complex, multifactorial phenotype. To the best of our knowledge, this is the first study to investigate the association between the rs10767664 and rs2030323 polymorphisms in the *BDNF* gene and nicotine use, as well as personality traits, in women. While the functional rs6265 polymorphism has been widely studied in relation to smoking behaviour and substance dependence, rs10767664 and rs2030323 have received considerably less attention, particularly in the context of nicotine or other substance dependencies. Our study expands the current knowledge of BDNF ’s potential role in addictive behaviours by exploring these understudied variants, offering a novel contribution to the field and paving the way for future research into their functional relevance in addiction-related phenotypes. The lower Agreeableness and Conscientiousness scores observed in women who use nicotine compared to never-smokers suggest that personality traits play a significant role in nicotine use in women. The observed relationship between personality traits and nicotine use lends support to the self-medication hypothesis, suggesting that some women may initiate or maintain nicotine use as a coping mechanism for stress and negative affect. Public health initiatives targeting women should consider personality and psychological risk factors in addition to biological risks, as these factors can also contribute to health outcomes. Our findings contribute to the understanding of nicotine dependence in women and highlight the importance of examining genetic and personality-related risk factors within a sex-specific context.

## Figures and Tables

**Table 1:** Hardy–Weinberg equilibrium for rs6265 in the cigarette users and never-smokers.

	Genotype	Observed	Expected	A Allele Frequency	G Allele Frequency	*p*-Value
Cigarette users *n* = 112	AA	1	1.63	27	197	0.5761
	AG	25	23.75			
	GG	86	86.63			
Never- smokers *n* = 127	AA	1	2.14	33	221	0.3693
	AG	31	28.71			
	GG	95	96.14			

*p*-value—statistical significance, *n*—number of subjects.

**Table 2:** Hardy–Weinberg equilibrium for rs10767664 in the cigarette users and never-smokers.

	Genotype	Observed	Expected	A Allele Frequency	T Allele Frequency	*p*-Value
Cigarette users *n* = 111	AA	1	2.31	32	190	0.3149
	AT	30	27.39			
	TT	80	81.31			
Never- smokers *n* = 121	AA	2	3.64	42	200	0.2972
	AT	38	34.71			
	TT	81	82.64			

*p*-value—statistical significance, *n*—number of subjects.

**Table 3:** Hardy–Weinberg equilibrium for rs2030323 in the cigarette users and never-smokers.

	Genotype	Observed	Expected	G Allele Frequency	T Allele Frequency	*p*-Value
Cigarette users *n* = 110	GG	79	80.33	188	32	0.3086
	GT	30	27.35			
	TT	1	2.33			
Never- smokers *n* = 121	GG	79	78.56	195	47	0.8001
	GT	37	37.87			
	TT	5	4.56			

*p*-value—statistical significance, *n*—number of subjects.

**Table 4:** Frequency of rs6265 genotypes and alleles in cigarette users and never-smokers.

	Genotypes			Alleles	
	AA *n* (%)	AG *n* (%)	GG *n* (%)	A *n* (%)	G *n* (%)
Cigarette users *n* = 112	1 (0.89%)	25 (22.32%)	86 (76.79%)	27 (12.05%)	197 (87.95%)
Never–smokers *n* = 127	1 (0.79%)	31 (24.41%)	95 (74.8%)	33 (12.99%)	221 (87.01%)
χ^2^ (*p*-value)	0.1495374 (0.92796)			0.0955 (0.7573)	

*n*—number of subjects, *p*—statistical significance, χ^2^—test statistics.

**Table 5:** Frequency of rs10767664 genotypes and alleles in cigarette users and never-smokers.

	Genotypes			Alleles	
	AA *n* (%)	AT *n* (%)	TT *n* (%)	A *n* (%)	T *n* (%)
Cigarette users *n* = 111	1 (0.9%)	30 (27.03%)	80 (72.07%)	32 (14.41%)	190 (85.59%)
Never–smokers *n* = 121	2 (1.65%)	38 (31.4%)	81 (66.94%)	42 (17.36%)	200 (82.64%)
χ^2^ (*p*-value)	0.8512681 (0.65336)			0.7471 (0.3874)	

*n*—number of subjects, *p*—statistical significance, χ^2^—test statistics.

**Table 6:** Frequency of rs2030323 genotypes and alleles in cigarette users and never-smokers.

	Genotypes			Alleles	
	GG *n* (%)	GT *n* (%)	TT *n* (%)	G *n* (%)	T *n* (%)
Cigarette users *n* = 110	79 (71.82%)	30 (27.27%)	1 (0.91%)	188 (85.45%)	32 (14.55%)
Never–smokers *n* =121	79 (65.29%)	37 (30.58%)	5 (4.13%)	195 (80.58%)	47 (19.42%)
χ^2^ (*p*-value)	2.880733 (0.23684)			1.9328 (0.1644)	

*n*—number of subjects, *p*—statistical significance, χ^2^—test statistics.

**Table 7:** NEO Five-Factor Inventory scores in cigarette users and never-smokers.

NEO Five-Factor Inventory	Cigarette Users (*n* = 112) M ± SD	Never–Smokers (*n* = 127) M ± SD	*p*-Value	Eta Squared (η^2^)	d_Cohen_
Neuroticism	5.893 ± 2.072	5.622 ± 1.877	0.255490	NA	NA
Extraversion	5.616 ± 2.085	5.299 ± 1.937	0.239026	NA	NA
Openness	5.411 ± 2.029	5.457 ± 2.011	0.798012	NA	NA
Agreeableness	5.446 ± 2.386	6.315 ± 2.329	0.005767 *	0.032	0.363
Conscientiousness	5.571 ± 2.061	6.882 ± 2.203	0.000012 *	0.08	0.591

*p*—statistical significance, M ± SD—mean ± Standard deviation, *—significant statistical difference, NA- not analysed.

**Table 8:** State–Trait Anxiety Inventory scores in cigarette users and never-smokers.

State-Trait Anxiety Inventory	Cigarette Users (*n* = 112) M ± SD	Never–Smokers (*n* = 127) M ± SD	*p*-Value
Trait anxiety	5.902 ± 2.352	5.606 ± 2.116	0.174650
State anxiety	5.598 ± 2.244	5.512 ± 2.243	0.653407

*p*—statistical significance, M ± SD—mean ± Standard deviation.

**Table 9:** Linkage disequilibrium of rs6265, rs10767664 and rs2030323 in the *BDNF* gene in groups of cigarette users and never-smokers.

		rs10767664	rs2030323
rs6265	D	0.1098	0.1092
	D′	0.9995	0.9995
	X^2^	357.9278	346.6917
	*p*-value	<2.2204 × 10^−16^	<2.2204 × 10^−16^
	N	229	229
rs10767664	D		0.1326
	D′		0.9838
	X^2^		429.3814
	*p*-value		<2.2204 × 10^−16^
	*n*		229

D′—Levontin’s standardised disequilibrium coefficient, x^2^—test statistics, *p*—statistical significance, *n*—number of subjects.

**Table 10:** Haplotype association analysis for the rs6265, rs107676664 and rs2030323 in the *BDNF* gene in a group of cigarette users and never-smokers.

Haplotype	Never–Smokers (*n* = 119)	Cigarette Users (*n* = 110)	*p*-Value
G A T	0.03356	0.02273	0.47669
A A T	0.13866	0.12273	0.59990
G T G	0.81087	0.85455	0.20167

*p*—statistical significance.

## Data Availability

Detailed genotyping and psychometric test results are available upon request.

## Abbreviations

The following abbreviations are used in this manuscript:

BDNF	Brain-derived neurotrophic factor
CNS	Central nervous system
D3R	Dopamine 3 receptor
DA	Dopamine
DR	Dopamine receptor
HWE	Hardy-Weinberg’s equilibrium
LD	Linkage disequilibrium
MDS	Mesolimbic dopaminergic system
MINI	Mini International Neuropsychiatric Interview
NAc	Nucleus accumbens
nAChRs	Nicotinic acetylcholine receptors
ND	Nicotine dependence
NEO-FFI	NEO Five-Factor Inventory
NMDA	N-Methyl-D-aspartate
p75NTR	p75 neurotrophin receptor
SD	Substance dependence
SNP	Single-nucleotide polymorphism
STAI	State-Trait Anxiety Inventory
TrkB	Tyrosine kinase receptor B
VTA	Ventral tegmental area
WHO	World Health Organisation
